# Light Regulation Under Equivalent Cumulative Light Integral: Impacts on Growth, Quality, and Energy Efficiency of Lettuce (*Lactuca sativa* L.) in Plant Factories

**DOI:** 10.3390/plants14223469

**Published:** 2025-11-13

**Authors:** Jianwen Chen, Cuifang Zhu, Ruifang Li, Zihan Zhou, Chen Miao, Hong Wang, Rongguang Li, Shaofang Wu, Yongxue Zhang, Jiawei Cui, Xiaotao Ding, Yuping Jiang

**Affiliations:** 1 Faculty of Urban Construction and Ecological Technology, Shanghai Institute of Technology, Shanghai 201418, China; 17689764503@163.com (J.C.);; 2 Shanghai Key Laboratory of Protected Horticultural Technology, Horticulture Research Institute, Shanghai Academy of Agricultural Sciences, Jinqi Road No. 1000, Fengxian District, Shanghai 201403, China; zcf@saas.sh.cn (C.Z.);

**Keywords:** lettuce, dynamic light regulation, energy use efficiency, nutritional quality

## Abstract

Facing the significant challenges posed by global population growth and urbanization, plant factories, as an efficient closed cultivation system capable of precise environmental control, have become a key direction in the development of modern agriculture. However, high energy consumption, particularly lighting (which accounts for over 50%), remains a major bottleneck limiting their large-scale application. This study systematically explored the effects of dynamic light regulation strategies on lettuce (*Lactuca sativa* L.) growth, physiological and biochemical indicators (such as chlorophyll, photosynthetic, and fluorescence parameters), nutritional quality, energy utilization efficiency, and post-harvest shelf life. Four different light treatments were designed: a stepwise increasing photosynthetic photon flux density (PPFD) from 160 to 340 μmol·m^−2^·s^−1^ (T1), a constant light intensity of 250 μmol·m^−2^·s^−1^ (T2), a three-stage strategy with high light intensity in the middle phase (T3), and a three-stage strategy with sequentially increasing light (T4). The results showed that the T4 treatment exhibited the best overall performance. Compared with the T2 treatment, the T4 treatment increased biomass by 23.4%, significantly improved the net photosynthetic rate by 50.32% at the final measurement, and increased ascorbic acid (AsA) and protein content by 33.36% and 33.19%, respectively. Additionally, this treatment showed the highest energy use efficiency. On the 30th day of treatment, the light energy use efficiency (LUE) and electrical energy use efficiency (EUE) of the T4 treatment were significantly increased, by 23.41% and 23.9%, respectively, compared with the T2 treatment. In summary, dynamic light regulation can synergistically improve crop yield, chlorophyll content, photosynthetic efficiency, nutritional quality, and energy utilization efficiency, providing a theoretical basis and solution for precise light regulation and energy consumption reduction in plant factories.

## 1. Introduction

Currently, traditional agriculture is facing global challenges, with population growth and rapid urbanization exerting immense pressure on horticultural production systems. Developing intensive and sustainable horticultural production models has become an urgent task [1]. The plant factory is a closed cultivation system that allows for precise regulation of environmental factors. With its green and sustainable characteristics, along with high levels of intensification, it has become a cutting-edge model in modern agriculture. However, high production and operational costs remain one of the major challenges limiting the development of plant factories. Reports indicate that energy costs dominate the total operating expenses of plant factories, with artificial lighting systems being a key subsystem contributing significantly to energy consumption, directly influencing crop productivity and resource use efficiency [2,3]. Currently, lighting energy consumption accounts for 60% to 80% of the total energy usage [3,4]. This phenomenon results in prices for vegetables produced in plant factories being significantly higher than those grown using traditional methods, thus limiting their large-scale commercialization. Therefore, improving the precision of light environment regulation to enhance light energy efficiency has become a critical issue that needs to be addressed in the plant factory field.

The light environment primarily consists of three factors: light quality, photosynthetic photon flux density (PPFD), and photoperiod. These factors collectively regulate crop morphogenesis, yield formation, and quality metabolism [5]. Previous studies have primarily focused on optimizing PPFD under fixed photoperiods or adjusting photoperiods under fixed PPFD conditions to explore the optimal lighting regime for crop growth [6,7,8]. However, such optimization strategies involving single variables are insufficient to fully analyze the plant’s adaptive responses to complex light environments and cannot effectively support the design of lighting formulas that simultaneously achieve the goals of “high yield, high quality, and energy efficiency”. The daily light integral (DLI), which refers to the total amount of photosynthetically active radiation received per unit area per day, is a key indicator for evaluating light energy supply [9]. Under the condition of maintaining a constant cumulative light integral (CLI) throughout the entire growth cycle, flexible combinations of PPFD and photoperiod can offer new pathways for optimizing crop light efficiency [10]. Existing studies have shown that, under constant CLI conditions, appropriate combinations of photoperiod and PPFD can significantly promote biomass accumulation in crops such as basil, pumpkin, and potato, while also reducing energy consumption [11,12,13]. In terms of lettuce (*Lactuca sativa* L.) light environment regulation, some progress has been made. For instance, Mao et al. [14] found that under the same CLI, using multiple PPFD combinations and extending the photoperiod promoted lettuce growth more effectively than using a constant PPFD. Yang et al. [15] through the use of a logistic model to segment growth stages and set differential DLI values, significantly increased the fresh weight of the aerial part and photon use efficiency. Jin et al. [16] employed a dynamic regulation method by increasing PPFD by 16 μmol·m^−2^·s^−1^ every three days. Although this approach did not significantly increase fresh weight, it did enhance dry matter accumulation and light energy utilization efficiency. However, existing studies primarily focus on optimizing fixed photoperiods or PPFD throughout the entire growth cycle. While some research has explored stage-specific dynamic light regulation, it has generally concentrated on growth parameters, overlooking its broader impacts. Therefore, this study proposes a comprehensive evaluation framework to systematically analyze the synergistic effects of dynamic lighting on lettuce, encompassing not only growth but also physiological and biochemical traits, post-harvest quality, and shelf life.

Lettuce, a biennial or annual herbaceous plant of the Asteraceae family, is one of the earliest domesticated vegetable species. Due to its short growth cycle, high economic value, and rich nutritional content, it has become an important leafy vegetable widely cultivated globally [17]. More importantly, this crop is highly sensitive to environmental factors, particularly light conditions, which significantly influence its growth, biomass formation, and nutritional quality (such as the accumulation of vitamins and polyphenols, as well as nitrate content). Key light parameters, including PPFD, photoperiod, and light quality, are crucial in regulating these aspects. Given these characteristics, lettuce is considered an ideal model crop for both commercial production in plant factories and light environment research [18].

Therefore, this study focuses on lettuce, aiming to explore the optimal production strategy under plant factory conditions. By systematically adjusting the combination of PPFD and photoperiod while maintaining the same CLI, we comprehensively analyze lettuce growth, physiological and biochemical traits, quality, energy utilization efficiency, and shelf life of post-harvest. This study stipulates that a successful optimization scheme must achieve a statistically significant improvement (*p* < 0.05) of at least 10% in at least one of the following primary success criteria, compared to the best fixed light intensity control: a ≥10% increase in light energy use efficiency (LUE) and electrical energy use efficiency (EUE), or a ≥10% increase in above-ground biomass. Additionally, ascorbic acid content is listed as a secondary observation indicator, and any increase in its level is considered a beneficial supplementary effect; however, it is not the primary criterion for determining the success of the scheme. The goal is to provide a theoretical foundation for efficient and high-quality lettuce production in plant factories and to offer technical references for reducing lighting energy consumption in plant factory operations.

## 2. Results

### 2.1. Effects of Different Treatments on Lettuce Growth and Development Under the Same CLI

**Figure 1:**
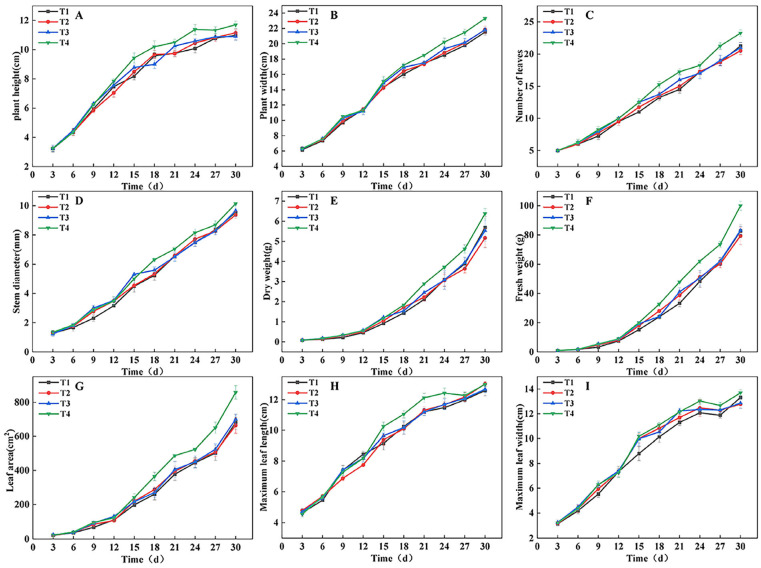
Trends in lettuce (*Lactuca sativa* L.) growth indicators under different light treatments. (**A**–**I**) Comparison of differences in plant height (**A**), plant width (**B**), number of leaves (**C**), stem diameter (**D**), dry weight (**E**), fresh weight (**F**), leaf area (**G**), maximum leaf length (**H**), and maximum leaf width (**I**) for each treatment every 3 d.

To monitor the effects of different light treatments (DLI strategies) on lettuce growth dynamics, growth parameters were measured every 3 days, and the results are shown in Figure 1. From the 9th day, significant differences were observed in plant height, plant width, number of leaves, stem diameter, fresh weight, dry weight, and leaf area (Figure 1A–G). In the early growth stage (around the 9th day), the T4 and T3 treatments performed better than the T2 and T1 treatments in most growth parameters. As the growing period progressed, the growth rate in the T3 treatment slowed in the middle stage but recovered in the later stage, and by the end of the experiment, its growth performance was similar to that of the T2 and T1 treatments. In contrast, the T4 treatment maintained superior growth throughout the entire growth period. At harvest (end of the experiment), leaf characteristic analysis showed that the maximum leaf length in the T1 treatment was lower than that of other treatments, while the maximum leaf width was greater in the T1 and T4 treatments than in the T2 and T3 treatments (Figure 1H,I).

**Figure 2:**
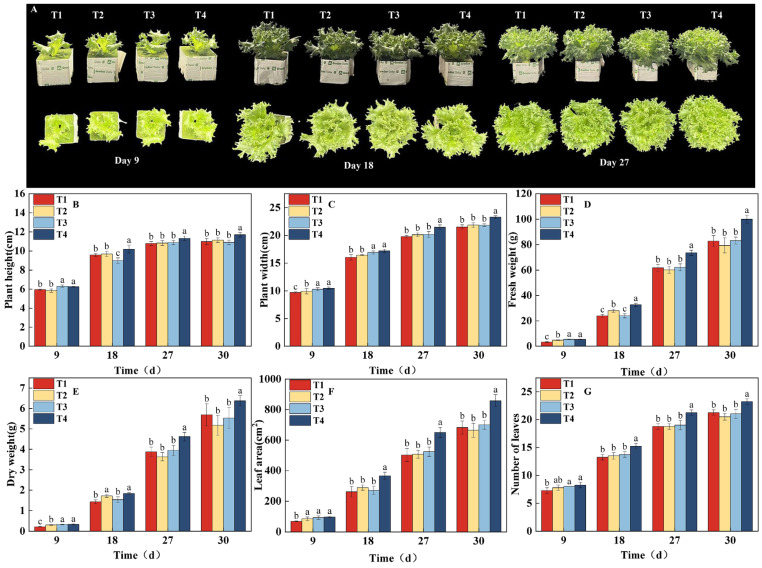
Effects of different light treatments on growth indicators and lettuce phenotype. (**B**–**G**) Comparison of differences in plant height (**B**), plant width (**C**), fresh weight (**D**), dry weight (**E**), leaf area (**F**), and number of leaves (**G**) for each treatment. (**A**) Phenotypic images taken every 9 d under different light treatments. Different letters in each graph indicate significant differences at *p* < 0.05.

The linear mixed model analysis revealed a significant “time × treatment” interaction for key growth traits, including plant width, fresh weight, dry weight, and leaf area, confirming that different light regulation strategies induce growth trajectories that fundamentally diverge over time (Table S1). This statistical evidence is corroborated by the results of the significance analysis. Based on the significance analysis of key growth parameters and growth stages (Figure 2), the differences between treatments became statistically significant from the 9th day. At this point, the T4 and T3 treatments showed significantly higher plant height, plant width, fresh weight, and dry weight compared to the T1 and T2 treatments (Figure 2B–E). As dynamic light regulation continued, the growth rate of the T3 treatment was notably suppressed in the middle stage, while the T4 treatment consistently maintained a growth advantage. The T1 treatment lagged behind in the early growth stages, but as the PPFD increased in a stepwise manner, its growth parameters gradually approached the levels of T2 and T3. By the harvest period (the 30th day), the T4 treatment significantly outperformed all other treatments in all measured parameters. Fresh weight reached 100.00 g, which was 20.85%, 25.94%, and 20.22% higher than that of T1 (82.75 g), T2 (79.40 g), and T3 (83.18 g), respectively. The dry weight was 6.38 g, which was 12.13%, 23.40%, and 15.37% higher than T1 (5.69 g), T2 (5.17 g), and T3 (5.53 g), respectively. The trends in leaf area and number of leaves were consistent with the above parameters (Figure 2F,G), with T4 showing a significant lead starting from the 18th day. Ultimately, leaf area reached 858.20 cm^2^, which was 25.99%, 29.29%, and 22.69% higher than T1 (681.16 cm^2^), T2 (663.75 cm^2^), and T3 (699.50 cm^2^), respectively. Combining the plant phenotypes shown in Figure 2A, it can be concluded that dynamic regulation of PPFD and photoperiod based on growth stages (as in the T4 treatment) can effectively promote crop growth and significantly enhance biomass accumulation. This is a feasible strategy for optimizing the light environment in plant factories to achieve high-yield and high-efficiency production.

### 2.2. Effects of Different Treatments on Lettuce SPAD, Photosynthetic Parameters, and Fluorescence Parameters Under the Same CLI

**Figure 3:**
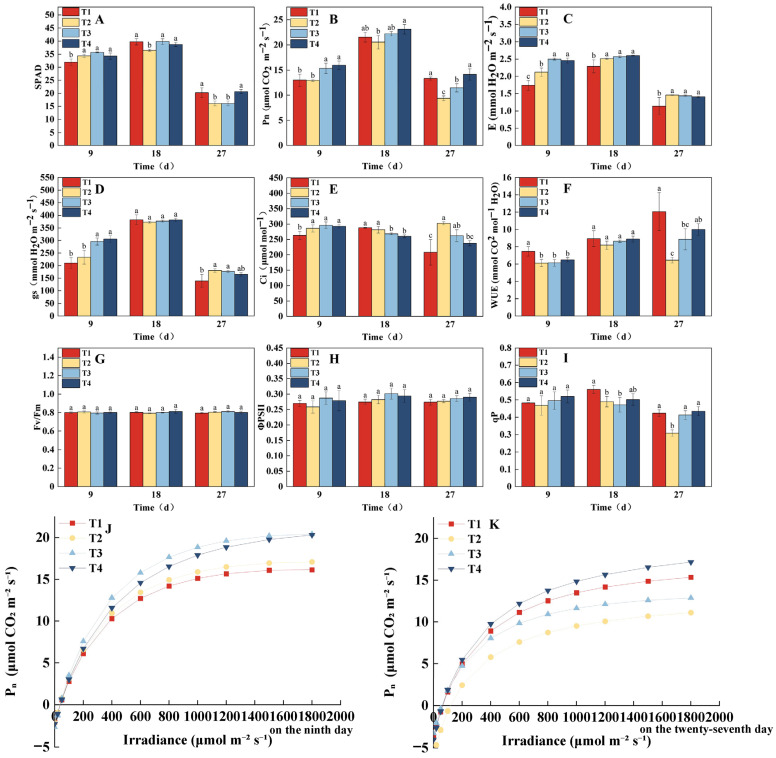
Effects of different light treatments on chlorophyll index, photosynthetic parameters, and chlorophyll fluorescence parameters of lettuce. (**A**–**I**) Comparison of differences in SPAD (**A**), Pn (**B**), E (**C**), gs (**D**), WUE (**E**), Ci (**F**), Fv/Fm (**G**), ΦPSII (**H**), and qP (**I**) for each treatment every 9 d. (**J**,**K**) Fitting of light response curves under different light treatments. Different letters in each graph indicate significant differences at *p* < 0.05.

The dynamic changes in SPAD and photosynthetic physiological characteristics of lettuce under different light treatments are shown in Figure 3. The SPAD generally followed an increasing trend initially, followed by a decline (Figure 3A), with significant differences observed between treatments at key growth stages. On the 9th day, the SPAD value in the T1 treatment was significantly lower than that in the other treatments. By the 18th day, the SPAD value of T2 reached its lowest point, and by the 27th day, the SPAD values in the T1 and T4 treatments were significantly higher than those in T2 and T3. Notably, the SPAD of T1 was lower in the early growth stage but significantly increased and even surpassed other treatments in the later stages. This could be attributed to the stepwise increase in PPFD (160 → 340 μmol·m^−2^·s^−1^), which likely promoted chlorophyll synthesis in the later growth stages. The T4 treatment consistently maintained the highest SPAD throughout the entire growth period, indicating that its photoperiod regulation scheme was the most favorable for the continuous accumulation and stable maintenance of chlorophyll. The photosynthetic parameters (Figure 3B–F) indicated that the T4 treatment consistently exhibited the highest Pn across all three measurement points. On the 9th day, Pn in the T4 and T3 treatments was significantly higher than in the T1 and T2 treatments. By the 18th day, Pn in the T4 treatment remained significantly higher than in T1 and T2, and by the 27th day, Pn in the T4 and T1 treatments was significantly higher than in T2 and T3, with Pn in T4 being 50.32% higher than in T2. The changes in E and gs followed a similar trend to Pn on the 9th day, with T3 and T4 significantly higher than T1 and T2. However, on the 18th and 27th days, the E value in the T1 treatment was significantly lower than that in the other treatments. No significant differences in gs were observed on the 18th day, but by the 27th day, gs in T1 was significantly lower than in T2 and T3. On the 9th and 27th days, WUE in T1 was significantly higher, while Ci was significantly lower compared to the other treatments, displaying opposite trends.

The light response curve analysis (Figure 3J,K) further indicated that on the 9th day, the maximum net photosynthetic rate (Pmax) was highest in the T3 and T4 treatments, reaching 20.44 and 20.32 μmol·m^−2^·s^−1^, respectively, which were significantly higher than those in T2 (17.10 μmol·m^−2^·s^−1^) and T1 (16.17 μmol·m^−2^·s^−1^). By the 27th day, Pmax was in the order of T4 (17.18) > T1 (15.35) > T3 (12.87) > T2 (11.11) μmol·m^−2^·s^−1^, which aligned with the trends observed in Pn (Figure 3B). In terms of chlorophyll fluorescence parameters (Figure 3G–I), there were no significant differences in Fv/Fm and ΦPSII between treatments throughout the growth period. However, qP was significantly higher in the T1 treatment on the 18th and 27th days compared to other treatments. In conclusion, the T4 treatment, which combined dynamic regulation of PPFD and photoperiod, optimized photosynthetic performance, promoted continuous accumulation of photosynthetic pigments, and maintained high light energy use efficiency, ultimately demonstrating the best and most stable overall effects.

### 2.3. Effects of Different Treatments on Lettuce Quality and Postharvest Shelf Life Under the Same CLI

**Figure 4:**
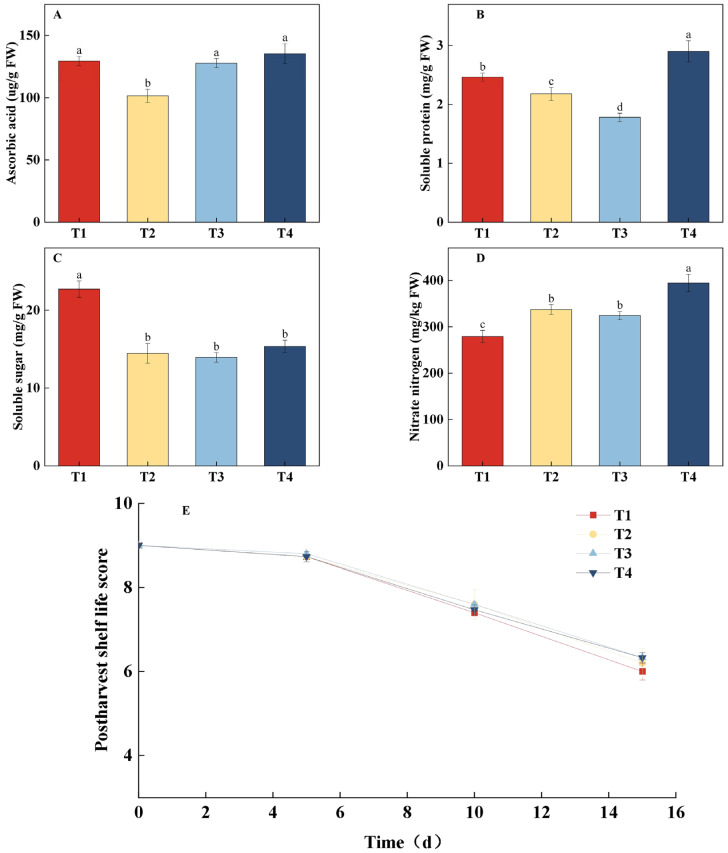
Effects of different light treatments on ascorbic acid (AsA) content, soluble protein content, soluble sugar content, nitrate nitrogen content, and shelf-life performance of lettuce. (**A**–**D**) Comparison of differences in AsA content (**A**), soluble protein content (**B**), soluble sugar content (**C**), and nitrate nitrogen content (**D**) for each treatment on the 30th day. (**E**) Fitting curve of the postharvest shelf life of lettuce under different light treatments. Different letters in each graph indicate significant differences at *p* < 0.05.

As shown in Figure 4, different treatments significantly affected lettuce quality. The T4 treatment resulted in the highest ascorbic acid (AsA) and soluble protein content, while the T2 treatment exhibited the lowest AsA content. T4 significantly increased AsA content by 33.36% compared to T2 (Figure 4A). Soluble protein content was lowest in the T3 treatment, and T4 significantly increased soluble protein content by 63.05% compared to T3 (Figure 4B). The results for soluble sugar and nitrate nitrogen content are shown in Figure 4C,D. The T1 treatment exhibited the highest soluble sugar content and the lowest nitrate nitrogen content, with no significant differences in soluble sugar content among the other treatments. The T4 treatment had the highest nitrate nitrogen content, which was significantly higher by 29.29% compared to T1. In terms of post-harvest shelf life (Figure 4E), there were no significant differences in sensory quality scores across all treatments on the 5th, 10th, and 15th days of storage. Comprehensive analysis indicated that the T4 treatment, which combined dynamic regulation of PPFD and photoperiod, effectively promoted the accumulation of AsA and soluble protein, but it also led to a significant increase in nitrate nitrogen content. In contrast, the T1 treatment, with its gradually increasing PPFD strategy, significantly enhanced soluble sugar content while reducing nitrate nitrogen accumulation. Additionally, all light treatments did not negatively affect post-harvest storage quality.

### 2.4. Effects of Different Treatments on Lettuce Energy Use Efficiency and Energy Consumption per Unit Yield Under the Same CLI

**Figure 5:**
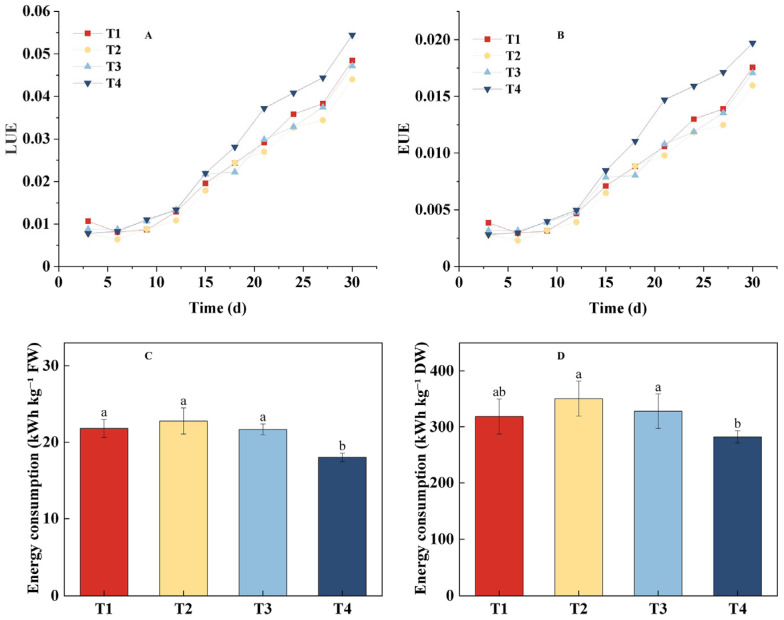
Effects of different light treatments on energy use efficiency and energy consumption per unit yield of lettuce. (**A**) Light use efficiency (LUE). (**B**) Energy use efficiency (EUE). (**C**) Energy consumption per unit fresh weight (kWh kg^−1^ FW). (**D**) Energy consumption per unit dry weight (kWh kg^−1^ DW). Different letters in each graph indicate significant differences at *p* < 0.05.

As shown in Figure 5, LUE and EUE exhibited similar trends across all treatments (Figure 5A,B). The T4 treatment gradually showed superior efficiency after the 15th day of growth and maintained this advantage until the end of the experiment. The energy use efficiency in the T1 treatment increased progressively with the PPFD and surpassed T2 and T3 treatments after the 15th day, ranking second only to T4 by the 30th day. The T3 treatment reached levels similar to T1 by the 21st day but was overtaken thereafter. The T2 treatment consistently showed the lowest efficiency throughout the growth period. By the 30th day, the efficiency rankings were as follows: T4 > T1 > T3 > T2. Specifically, the LUE and EUE of the T4 treatment were 0.0543 and 0.0197, respectively, which were significantly higher than the T2 treatment (0.044 and 0.0159), with improvements of 23.41% and 23.9%, respectively. The analysis of energy consumption per unit yield provided further insights (Figure 5C,D). For fresh weight production (kWh kg^−1^ FW), the T4 treatment was the most efficient, consuming significantly less energy than T1, T2, and T3. In contrast, for dry matter production (kWh kg^−1^ DW), the results were more nuanced: while T4 and T1 demonstrated statistically equivalent energy efficiency, T4 was significantly more energy-efficient than both T2 and T3. In conclusion, the dynamic regulation strategy combining PPFD and photoperiod in the T4 treatment showed significant advantages in improving both light and energy use efficiencies.

### 2.5. Correlation Analysis of Plant Morphology, Light Environment, and LUE/EUE

**Figure 6:**
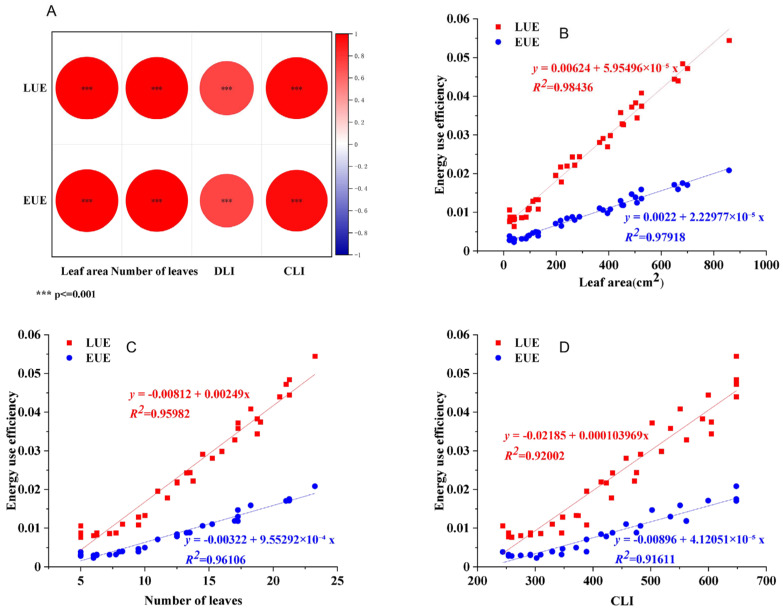
Association analysis of plant morphology, light environment with LUE and EUE. (**A**) Correlation analysis diagram of leaf area, number of leaves, DLI and CLI. (**B**–**D**) Fitting curves of leaf area, number of leaves and CLI with energy use efficiency.

To quantify the relationship between energy use efficiency (LUE/EUE) and key growth indicators as well as light environment parameters, a Pearson correlation analysis was conducted (Figure 6A). Subsequently, linear regression models were fitted for the variables showing high correlation with LUE/EUE—namely, number of leaves, leaf area, and CLI—to describe their predictive relationships (Figure 6B–D). Residual analysis results verified the model assumptions (Figure S1). The results revealed significant positive linear relationships between LUE/EUE and leaf area, leaf number, and CLI, indicating that higher values of these structural and environmental traits were associated with improved energy use efficiency. It is worth noting that the T4 treatment showed the largest leaf area and the most leaves among all treatments, which aligns with its highest recorded LUE/EUE values. These results suggest that the superior energy use efficiency observed in the T4 treatment was correlated with a canopy structure possessing a greater total leaf area and more leaves—traits that may reflect enhanced light interception and utilization.

## 3. Discussion

PPFD and photoperiod are two key environmental factors that regulate plant growth and development. Even under the same DLI, different combinations of PPFD and photoperiod can significantly affect plant growth [15]. Numerous studies have shown that, compared with static light strategies, dynamic light strategies, while maintaining consistent CLI, can significantly increase crop yield and reduce energy consumption [11,12,13,16]. This is mainly due to the varying light environment requirements of plants at different growth stages; a constant DLI may result in excess light energy during early stages and insufficient light in later stages, thus reducing light use efficiency [19]. Previous research has identified 14.4 mol·m^−2^·d^−1^ as a critical DLI value that optimizes both plant yield and resource use efficiency [20,21]. In this study, the 16 h photoperiod and 250 μmol·m^−2^·s^−1^ PPFD treatment (T2) was used as a control, which had a DLI of 14.4 mol·m^−2^·d^−1^, while the CLI of the other three dynamic light treatments (T1, T3, T4) was kept consistent with this control. Experimental results indicated that the T4 treatment, which dynamically adjusted light, showed the best performance. Notably, the light environment parameters in T4 and T3 treatments were identical for the first 10 days (PPFD 200 μmol·m^−2^·s^−1^, photoperiod 17.5 h). On the 9th day, both treatments exhibited superior growth compared to other treatments. This could be due to the combination of a longer photoperiod and a weaker PPFD, which may promote the accumulation of photosynthetic products [22]. During the period from the 11th to the 20th day, T4 used a moderate PPFD (250 μmol·m^−2^·s^−1^) along with a longer photoperiod (16 h). This strategy was effective in avoiding the light inhibition caused by high PPFD and promoted continuous accumulation of assimilates by extending the photosynthetic period [23]. In the later growth stages (the 21st to 30th day period), lettuce entered a rapid growth phase, with canopy closure and peak leaf photosynthetic capacity. During this period, T4 provided the highest PPFD (300 μmol·m^−2^·s^−1^), fully matching the plant’s photosynthetic potential, thereby exhibiting the highest Pn and Pmax, and significantly promoting biomass accumulation [15]. In contrast, T3 reduced the PPFD to 250 μmol·m^−2^·s^−1^ during this critical period, which failed to meet its maximum photosynthetic potential and limited further biomass accumulation. While T1 used a progressively increasing PPFD strategy, its lower initial PPFD in the early stages may have restricted early growth rates, resulting in a final biomass accumulation that did not surpass that of T4. The final fresh weight of T1 was not significantly different from that of T2 (fixed light regimen), consistent with the findings of Jin et al. [16].

SPAD, measured by the absorbance of leaf tissue at specific wavelengths, indirectly reflect the relative concentration of chlorophyll per unit leaf area. Higher SPAD readings typically indicate greater light energy capture capability and photosynthetic potential [24]. The results of this study support this notion, with the T4 treatment consistently maintaining higher SPAD throughout the growth period, alongside the highest levels of Pn and Pmax. Notably, by the 27th day, the T1 treatment showed no significant difference in SPAD and Pn from T4 as PPFD gradually increased, demonstrating enhanced photosynthetic performance with the optimized light environment. Photosynthesis is the core process in which green plants convert light energy into chemical energy, a process crucial to plant productivity and finely regulated by external environmental factors [25,26]. Studies have shown that although lower PPFD may limit the operation of photosynthetic apparatus, it can still maintain stable energy supply, supporting carbon assimilation and other metabolic processes [27]. In this study, the T4 and T3 treatments, which used lower PPFD in combination with longer photoperiods in the early stages, showed significantly higher Pn, indicating that this combination effectively promoted photosynthesis while ensuring a continuous energy supply. As leaves develop, photosynthetic rates often decline, which is related both to morphological and structural changes and to the decreased activity of the Rubisco enzyme [15,28]. This study also found that Pn declined from its peak on the 18th day to a lower value by the 27th day. Throughout the growth stages, the Fv/Fm ratio of all treatments remained stable at approximately 0.8, with no significant differences between treatments, suggesting that the light environments did not cause light inhibition or stress [29,30]. The qP parameter, which reflects the proportion of light energy allocated to photochemical reactions and the open state of photosystem II reaction centers, provides insight into light energy use efficiency [31]. The results showed no significant difference in qP between treatments on the 9th day; however, by the 18th day, the qP value for the T3 treatment had significantly decreased, while T1 and T4 maintained higher levels. The decline in qP in the T3 treatment is likely due to the sudden shift to high PPFD (300 μmol·m^−2^·s^−1^) from the 11th to the 20th day, which exceeded the short-term adaptive capacity of the plant. This imbalance between light absorption and carbon assimilation may have led to increased closure of reaction centers and a reduction in qP, with the excess light energy likely being dissipated primarily as heat through processes such as non-photochemical quenching (NPQ) [32]. Although this is a light-protective mechanism, its energy use efficiency is much lower than that of photochemical reactions. By the 27th day, both T1 and T4 maintained higher qP values, consistent with their higher Pn at the same time, suggesting that their photochemical reactions and carbon assimilation were efficiently coordinated, leading to more effective light energy conversion.

Leafy vegetables, such as lettuce, are significantly influenced by environmental factors such as light, which plays a crucial role in determining their quality [33]. Therefore, a systematic study of the impact of light treatments on the quality of these vegetables is of great importance. In this study, the AsA content in the T2 treatment was significantly lower than in other treatments. From the perspective of light environment settings, both T1 and T4 treatments maintained light intensities above 300 μmol·m^−2^·s^−1^ in the later growth stages, with T3 also reaching this PPFD during the mid-growth period, while T2 consistently maintained 250 μmol·m^−2^·s^−1^. Previous studies have shown that higher PPFD enhances overall photosynthetic capacity, thus promoting the synthesis and accumulation of AsA [34,35], which could be one reason for the lower AsA content observed in T2. The protein content was highest in the T4 treatment, significantly superior to other treatments, which may be attributed to higher PPFD enhancing enzyme activity related to protein synthesis [8]. Furthermore, the T1 treatment showed the highest soluble sugar content and the lowest nitrate nitrogen content. The gradual increase in PPFD in T1 may have better coordinated the carbon-nitrogen metabolic balance, promoting the distribution of photosynthetic products towards nitrogen absorption and conversion, thereby improving nitrogen use efficiency, reducing nitrate accumulation, and increasing soluble sugar content. Previous research has also indicated that lower PPFD promotes nitrate accumulation, while higher PPFD helps reduce nitrate content [5,36].

In plant factories, reducing energy consumption and increasing crop yield are considered two key goals for development [37]. To achieve high-quality and high-yield production of different crop varieties, light environment regulation strategies that align with their physiological characteristics must be formulated. Particularly, dynamic lighting schemes that respond to the unique needs of individual plants should be developed to significantly reduce system energy consumption while improving production efficiency [8]. In this study, energy use efficiency was highest in the T4 treatment, followed by T1, T3, and T2. The T1 treatment adopted a stepwise increase in PPFD, maintaining PPFD above 300 μmol·m^−2^·s^−1^ after 21 days of growth. This strategy significantly increased the DLI, providing sufficient energy for photosynthesis and promoting carbon assimilation rates beyond the plant’s growth requirements. Excess photosynthetic products accumulated as carbohydrates in the tissues, thus increasing dry matter content. This physiological process is typically accompanied by cell wall thickening, tighter cell structures, and enhanced storage material content, such as soluble sugars and proteins [38,39]. The energy consumption per unit fresh weight (kWh kg^−1^ FW) is directly relevant to post-harvest operations, as it determines the resource expenditure for handling, packaging, and refrigerated transport per unit of marketable yield. In contrast, the energy consumption per unit dry weight (kWh kg^−1^ DW) serves as an indicator of the intrinsic conversion efficiency from electrical energy into photosynthetic assimilates, reflecting the system’s carbon fixation capacity. In this study, the superior performance of the T4 treatment in both metrics suggests that this strategy is not only physiologically efficient but also has significant potential for reducing energy costs in practical applications. Shelf life determination is a critical link connecting agricultural production with market demand. In this study, no significant differences were observed in the shelf life performance of the different treatments, suggesting that the various light treatments did not negatively affect the post-harvest storage tolerance of lettuce. Although some studies have suggested that high PPFD before harvest may help extend shelf life [40], no such effect was observed in this study. Future research that identifies a light treatment capable of both reducing energy consumption and significantly extending shelf life would greatly enhance its practical application value, as it directly relates to the realization of economic benefits.

Regarding fixture efficacy, although there are variations in the photosynthetic photon efficacy of commercial LEDs, these variations are expected to have a systemic effect across all treatments. Less efficient light fixtures lead to higher energy consumption for the same photon flux, thus increasing energy consumption per unit yield [41]. However, since the T4 treatment demonstrates inherent advantages in light-to-biomass conversion efficiency, as evidenced by its significantly improved energy use efficiency and biomass yield, it is anticipated that T4’s relative energy efficiency advantage will remain consistent even under varying fixture efficacy conditions. This suggests that the T4 treatment is robust against common fluctuations in fixture efficacy, particularly with regard to the key energy efficiency metric of kWh per kg. In contrast, the study’s conclusions are more sensitive to changes in spectral power distribution (SPD). The optimization results were obtained under a specific red-to-blue light ratio, as spectral composition, particularly the red-to-blue ratio, is a critical environmental signal influencing plant morphological development [42]. It can be inferred that the advantages of T4 treatment would persist under other spectral compositions that promote canopy development and leaf expansion. However, if the spectral composition were to change significantly, such as through a substantial increase in the blue light ratio (known to inhibit leaf elongation), the canopy structure advantage of T4 treatment could be diminished, potentially affecting its energy efficiency ranking [43]. In summary, this study demonstrates that the T4 treatment proved to be a more energy-efficient option under the 8:2 red-to-blue light ratio and similar spectral characteristics.

This study has certain limitations and provides insights into future research directions. Some chlorophyll fluorescence parameters, such as NPQ, were not measured, which limits our ability to comprehensively analyze the impact of different light treatments on the energy dissipation mechanisms of the photosystem. Additionally, quality indicators were assessed only once at the harvest stage, despite the dynamic regulation of the light environment throughout the growth cycle. Future studies could synchronize the monitoring of quality parameters at multiple growth stages to more accurately elucidate the temporal relationship between light regulation and quality development. The study also employed a staged nutrient solution electrical conductivity (EC) management strategy, with EC values gradually increasing from 1.3 to 2.0 mS·cm^−1^. While this uniform EC management approach ensured valid comparisons between treatments, potential interactions between nutrient management and the light environment warrant further investigation in future experiments. It should be noted that CO_2_ enrichment technology was not employed in this study. However, future research incorporating this technique is expected to achieve a significant increase in yield. The main conclusions, particularly the assertion that the dynamic light control strategy (T4) is superior, were validated under specific experimental conditions, including the use of the ‘You Ya’ lettuce cultivar, an 8:2 red-to-blue light ratio, a temperature of 22 ± 2 °C, relative humidity of 70–80%, and a gradual increase in nutrient solution EC (from 1.3 to 2.0 mS·cm^−1^). Given these specific conditions, caution should be exercised when generalizing the conclusions to other cultivars, light quality formulations, environmental conditions, or nutrient management strategies. Pilot verification in diverse settings is recommended. To facilitate the practical application of the findings, we have summarized the optimized T4 strategy into a clear and actionable three-phase lighting management plan (Table 1). This plan provides detailed lighting parameters for each growth stage, offering a direct reference for practical production in plant factories.

## 4. Materials and Methods

### 4.1. Experimental Materials and Cultivation Environment

**Figure 7:**
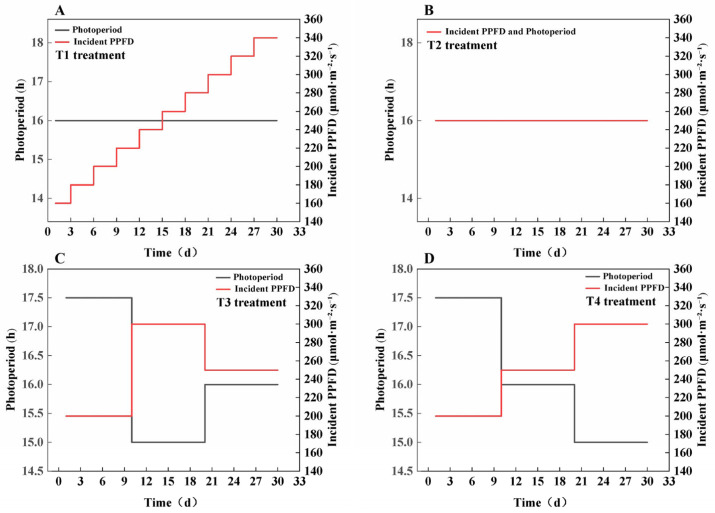
Schematic diagram of all light treatments. (**A**) T1, (**B**) T2, (**C**) T3, (**D**) T4.

This study used the lettuce variety ‘You Ya’ as the experimental material and was conducted at the Plant Factory of the National Engineering Research Center for Facility Agriculture, Chongming Base (31.34° N, 121.41° E). Prior to the formal treatment, seeds were sown into 240-cell trays containing rock wool and cultivated under a 16 h/8 h photoperiod (light/dark) with a PPFD of 250 μmol·m^−2^·s^−1^ and a red-to-blue light ratio of 8:2 for 15 days, until the plant height reached 3.3 cm. The seedlings were then transplanted into the experimental treatment groups. This study included four light treatments, each with four small subplots as replicates, all maintaining the same CLI and a consistent red-to-blue light ratio of 8:2 throughout the experimental period. In the T1 treatment, a stepwise increase in PPFD was applied, starting with an initial PPFD of 160 μmol·m^−2^·s^−1^ and a 16 h photoperiod, with the PPFD increasing by 20 μmol·m^−2^·s^−1^ every three days until it reached 340 μmol·m^−2^·s^−1^. The T2 treatment involved a constant PPFD of 250 μmol·m^−2^·s^−1^ and a constant photoperiod of 16 h. The T3 treatment featured a three-stage light regulation strategy, with Days 1–10 at 200 μmol·m^−2^·s^−1^ and a 17.5 h photoperiod, Days 11–20 at 300 μmol·m^−2^·s^−1^ and a 15 h photoperiod, and Days 21–30 at 250 μmol·m^−2^·s^−1^ and a 16 h photoperiod. The T4 treatment also applied a three-stage strategy, with Days 1–10 at 200 μmol·m^−2^·s^−1^ and a 17.5 h photoperiod, Days 11–20 at 250 μmol·m^−2^·s^−1^ and a 16 h photoperiod, and Days 21–30 at 300 μmol·m^−2^·s^−1^ and a 15 h photoperiod. A schematic diagram of all light treatments is shown in Figure 7, and the detailed PPFD spectra are presented in Figure S2. To ensure precise and stable light control, the PPFD at the canopy level was monitored regularly using a hand-held spectrometer (ALP-01, Shanghai Hesheng Instrument Technology Co., Ltd., Taiwan, China). The light fixture output was dynamically adjusted based on these measurements to consistently maintain the target PPFD for each treatment.

The environmental parameters in the plant factory were set as follows: temperature was maintained at 22 ± 2 °C, relative humidity (RH) ranged from 70% to 80%, and CO_2_ concentration was kept at natural levels. The plants were irrigated with nutrient solution every 2–3 days. The pH of the nutrient solution was kept constant between 5.5 and 6.0. The EC of the nutrient solution followed a stage-specific increment strategy: from Days 1–5, the EC was set at 1.3 mS·cm^−1^; from Days 6–10, it increased to 1.5 mS·cm^−1^; from Days 11–15, it was raised to 1.8 mS·cm^−1^; and thereafter, it was maintained at a stable level of 2.0 mS·cm^−1^. (The composition of the nutrient solution is shown in Table 2).

### 4.2. Measurement of Growth Parameters

Each treatment consisted of four subplots, serving as replicates. Destructive sampling occurred every three days, with one plant randomly selected from each subplot for measurement. The parameters assessed included plant height, plant width, leaf number, maximum leaf length, maximum leaf width, leaf area, stem diameter, fresh weight, and dry weight. The specific methods for measurement were as follows: plant height was recorded from the surface of the rock wool substrate to the highest point of the plant’s canopy using a tape measure; plant width was determined by measuring the maximum diameter of the aerial parts of the plant with a tape measure; leaf area was analyzed using ImageJ software (version 1.54g); the maximum leaf length and width were measured with a tape measure; stem diameter was taken using a vernier caliper; and fresh and dry weights were measured with an electronic analytical balance, focusing on the aerial biomass only.

### 4.3. Measurement of Chlorophyll and Carotenoid Content, Leaf Photosynthetic Rate, and Chlorophyll Fluorescence

The relative chlorophyll content, represented as SPAD, was measured in plant leaves using a portable SPAD meter (HPL-B3, Shanxi Hanpu Xun Optoelectronic Technology Co., Ltd., Shanxi, China). Photosynthetic and chlorophyll fluorescence parameters were assessed with the CIRAS-3 portable photosynthesis system (PP Systems, Amesbury, MA, USA). The parameters measured included net photosynthetic rate (Pn), intercellular CO_2_ concentration (Ci), stomatal conductance (Gs), transpiration rate (Tr), water-use efficiency (WUE), effective quantum yield of PSII (ΦPSII), and photochemical quenching coefficient (qP). During measurements, the leaf chamber environmental parameters were strictly maintained at the following setpoints to standardize conditions: leaf temperature was set at 25 °C, the leaf-to-air vapor pressure deficit (VPD leaf) was stabilized at 1.1 ± 0.1 kPa, the CO_2_ concentration was approximately 400 μmol mol^−1^, and the chamber was equipped with an internal 1000 PARi illumination unit. In addition, the maximum photochemical efficiency of PSII (Fv/Fm) was measured using the Plant Efficiency Analyzer (PEA, Hansatech Instruments Ltd., King’s Lynn, UK) after a 30 min dark adaptation. All measurements were conducted on the 9th, 18th, and 27th days.

### 4.4. Measurement of Soluble Sugars, Soluble Proteins, Ascorbic Acid and Nitrite Nitrogen

On the 30th day, four lettuce plants from each treatment group were randomly selected for sampling. The samples were immediately immersed in liquid nitrogen for rapid freezing and transferred to a −80 °C ultra-low temperature freezer for storage until subsequent physiological and biochemical analysis. The soluble sugar content was measured using the anthrone colorimetric method [44], soluble protein content was measured by the Coomassie brilliant blue method [45], ascorbic acid content was determined by the indole-3-acetic acid colorimetric method [46], and nitrate nitrogen content was measured using the salicylic acid colorimetric method [47]. All four parameters were tested using commercially available kits (Suzhou Kemin Biotechnology Co., Ltd., Suzhou, China).

### 4.5. Calculation of Energy Use Efficiency and Light Integral

LUE and EUE were calculated using the following equations [48,49].

(1) $$\mathrm{LUE}=\frac{\mathrm{fD}}{\mathrm{PAR}}$$

(2) $$\mathrm{EUE}=\frac{\mathrm{fD}}{E}$$

*PAR* (MJ/m^2^) = PPFD × 0.2176 × (Photoperiod × 3600) × Days/1,000,000 (3)

*E* (kWh) = Total power (kW) × Total operation hours (4)

In the above equations, *fD* represents the chemical energy per unit dry matter (20 MJ/kg); *D* refers to the dry matter increment per unit cultivation area (kg/m^2^); *PAR* refers to the cumulative photosynthetically active radiation during cultivation (MJ/m^2^). *E* represents the cumulative electrical energy input per unit cultivation area (MJ/m^2^). Each cultivation unit was equipped with five LED tubes. When operating at full capacity, each tube had a rated power of 30 W and provided a PPFD of 250 μmol·m^−2^·s^−1^. The power consumption of other lighting systems was estimated. The corresponding lighting and energy input data for each treatment protocol throughout the growth cycle are provided in Table S3.

In this study, both phases were conducted in a plant factory; therefore, the DLI and CLI were calculated.

DLI (mol·m^−2^·d^−1^) = PPFD × Photoperiod duration × 3600 × 10^−6^ (5)

CLI (mol·m^−2^) = PPFD × Cumulative light duration × 3600 × 10^−6^ (6)

### 4.6. Shelf Life Evaluation of Postharvest

On the 30th day at harvest, three lettuce plants were randomly selected from each light treatment group. Three functional leaves from each plant were collected and placed in a standardized circular plastic box (diameter: 18 cm, height: 7.3 cm). For each light treatment, three sample boxes were established as replicates. Two pieces of thoroughly moistened filter paper were placed at the bottom of each box to maintain a high-humidity environment. To ensure gas exchange, seven ventilation holes (approximately 1–2 mm in diameter) were evenly drilled in the cover of each box. The plastic boxes were then stored in the dark at 4 °C. Visual quality assessments were carried out on the 5th, 10th, and 15th days of storage. The overall visual quality score followed the method of Min et al. [40]. At each sampling time point, the leaves from three replicate samples (i.e., three plastic boxes) from each treatment were assessed according to the criteria shown in Table S2 (based on Min et al. [20] using a 9-point scale (where 9 represents the best quality and 1 represents the worst quality).

### 4.7. Statistical Analysis

Data analysis was performed using Excel 2021 and SPSS 25 for statistical analysis. Graphs were generated using Origin 2021. The interaction model was calculated using the R software (version 4.5.1). One-way analysis of variance (ANOVA) was used for significance testing, with a statistical significance level set at *p* < 0.05.

## 5. Conclusions

This study demonstrated that the dynamic light regulation strategy (T4) promoted plant growth, improved energy use efficiency, and optimized quality in a controlled plant factory environment. Compared to the static light treatment (T2), the T4 strategy significantly increased biomass, LUE, and EUE by 23.51% (95% CI [10.72, 36.30]), 23.41% (95% CI [10.60, 36.22]), and 23.90% (95% CI [10.82, 36.98]), respectively. In terms of quality, although the T4 treatment led to an increase in nitrate nitrogen content in the leaves to 394.66 ± 18.8 mg/kg, this level is well below the national food safety standard (limit of 3000 mg/kg), and it was accompanied by significant improvements in protein and AsA content. Therefore, the dynamic light regulation strategy not only promoted plant growth and improved energy use efficiency, but also optimized quality, providing a safe and efficient technical pathway for precise light management in controlled-environment agriculture.

## Figures and Tables

**Table 1:** Three-phase lighting plan.

Growth Stage	PPFD (μmol m^−2^ s^−1^)	Photoperiod (h)	Phase Objective & Expected Outcome
Stage I: Canopy Establishment (Days 1–10 after transplanting)	200 ± 5	17.5	Objective: To promote rapid leaf expansion and canopy formation. Outcome: Establishes sufficient photosynthetic area, laying the foundation for high yield.
Stage II: Biomass Accumulation (Days 11–20)	250 ± 5	16	Objective: To balance photosynthesis and respiration for efficient biomass accumulation. Outcome: Maintains vigorous growth momentum and optimizes the conversion efficiency of light to biomass.
Stage III: Yield Maximization (Days 21–30 until harvest)	300 ± 5	15	Objective: To maximize the synthesis and accumulation of photosynthetic assimilates. Outcome: Achieves the highest fresh weight yield, while the reduced photoperiod partially offsets the increased energy consumption from higher PPFD.

Note: Expected Energy Performance: Under the conditions of this study, the implementation of this strategy is expected to increase biomass by approximately 23.51% (95% CI [10.72, 36.30]) and reduce electrical energy consumption per unit yield (kWh kg^−1^ DW) by approximately 19.45% (95% CI [−30.99, −7.91]), compared to conventional static lighting regimes.

**Table 2:** The nutrient solution formula for lettuce cultivation.

Fertilizer Name	Units (mg/L)	Fertilizer Name	Units (mg/L)
5Ca(NO_3_)_2_·NH_4_NO_3_·10H_2_O	1188.78	MnSO_4_·H_2_O	2.13
NH_4_H_2_PO_4_	132.284	Na_2_B_4_O_7_·10H_2_O	4.41
MgSO_4_·7H_2_O	246.47	ZnSO_4_·7H_2_O	0.22
KNO_3_	369	CuSO_4_·5H_2_O	0.08
K_2_SO_4_	121.968	(NH_4_)_6_Mo_7_O_24_·4H_2_O	0.02
EDTA-Fe	40		

## Data Availability

The original contributions presented in this study are included in the article/Supplementary Material. Further inquiries can be directed to the corresponding author.

## Supplementary Materials

The following supporting information can be downloaded at: https://www.mdpi.com/article/10.3390/plants14223469/s1, Figure S1. Different PPFD spectrum diagrams. Figure S2. Regular residuals of the linear models for plant morphology, light environment, LUE, and EUE. Table S1: Significance test of time × treatment interaction effects. Table S2: Postharvest Shelf-Life Assessment of Lettuce. Table S3: Lighting and energy inputs for each treatment across the growth cycle. Supplementary 2: Statistical results of intergroup comparisons of plant growth metrics.
