# Supplementary material for: Light Regulation Under Equivalent Cumulative Light Integral: Impacts on Growth, Quality, and Energy Efficiency of Lettuce (Lactuca sativa L.) in Plant Factories
Source: Plants (Basel). 2025 Nov 13;14(22):3469. doi: 10.3390/plants14223469 (PMC12655942; doi:10.3390/plants14223469)
Supplement: Supplementary file 1 [file plants-14-03469-s001.zip › Supplementary S1.pdf]

Table S1 Significance test of time × treatment interaction effects.

Metric	Interaction_p_value	Significance
Plant height	0.3547	ns
Plant width	0.0009	***
Leaf number	< 0.0001	***
Maximum leaf length	0.2979	ns
Maximum leaf width	0.5699	ns
Fresh weight	0.0018	**
Dry weight	0.0262	*
Stem diameter	0.0052	**
Leaf area	< 0.0001	***

Table S2 Postharvest shelf-life assessment of lettuce.

Grade	*Appearance*	*Texture*	*Odor*
9- Excellent	Bright, fresh green. No yellowing/browning. Fresh cut edge.	Firm and crisp.	Fresh, grassy notes.
8- Very good	Bright, typical green. Slight leaf yellowing. Minor cut edge browning.	Firm and crisp.	Pronounced grassy aroma.
7- Good	Green with some yellow leaves. No noticeable cut edge browning.	Firm and crisp.	Weakened, no off-odor.
6- Satisfactory	Faded green with yellowing. Localized browning. Moderate cut edge browning.	Slight softening.	No off-odor.
5- Mediocre	Faded green, multiple yellow leaves. Dark, moist cut edge.	Softened.	Slight fermented odor.
4- Borderline	Noticeable discoloration. Cut edge with exudate.	Significantly softened.	Noticeable fermented odor.
3- Poor	Severe discoloration (brown). Moist tissue. Water exudation.	Significantly softened.	Sour/decaying notes.
2- Bad	Yellow/white or necrotic leaves. Soft, watery cut edge.	Mushy, no elasticity.	Strong rancid/putrid smell.
1- Very bad	Complete discoloration and necrosis. Rotten tissue.	Texture completely lost.	Extremely putrid smell.

Table S3 Lighting and energy inputs for each treatment across the growth cycle.

Treatment	Time(d)	PAR（MJ/m²）	E（kWh）	PPFD（μmol·m^{-2}·s^{-1}）	LED power（w）
T1	1—3	6.000 ± 0.108	4.608 ±0.083	160±5	19.2±0.6w
	4—6	6.750 ± 0.108	5.184 ± 0.083	180±5	21.6±0.6w
	7—9	7.500 ± 0.108	5.760 ±0.083	200±5	24±0.6w
	10—12	8.250 ± 0.108	6.336 ±0.083	220±5	26.4±0.6w
	13—15	9.000 ± 0.108	6.912 ± 0.083	240±5	28.8±0.6w
	16—18	9.750 ± 0.108	7.488 ±0.083	260±5	31.2±0.6w
	19—21	10.500 ± 0.108	8.064 ± 0.083	280±5	33.6±0.6w
	22—24	11.250 ± 0.108	8.640 ± 0.083	300±5	36±0.6w
	25—27	12.000 ± 0.108	9.216 ± 0.083	320±5	38.4±0.6w
	28—30	12.750 ± 0.108	9.792 ±0.083	340±5	40.8±0.6w
T2	1—3	9.375 ± 0.108	7.200 ± 0.083	250±5	30±0.6w
	4—6	9.375 ± 0.108	7.200 ± 0.083	250±5	30±0.6w
	7—9	9.375 ± 0.108	7.200 ± 0.083	250±5	30±0.6w
	10—12	9.375 ± 0.108	7.200 ± 0.083	250±5	30±0.60w
	13—15	9.375 ± 0.108	7.200 ±0.083	250±5	30±0.6w
	16—18	9.375 ± 0.108	7.200 ±0.083	250±5	30±0.6w
	19—21	9.375 ± 0.108	7.200 ± 0.083	250±5	30±0.6w
	22—24	9.375 ± 0.108	7.200 ± 0.083	250±5	30±0.6w
	25—27	9.375 ± 0.108	7.200 ± 0.083	250±5	30±0.6w
	28—30	9.375 ± 0.108	7.200 ±0.083	250±5	30±0.6w
T3	1—3	8.203 ± 0.118	6.300 ± 0.091	200±5	24w±0.6w
	4—6	8.203 ± 0.118	6.300 ± 0.091	200±5	24w±0.6w
	7—9	8.203 ± 0.118	6.300 ± 0.091	200±5	24w±0.6w
	10—12	9.765 ± 0.107	7.500 ± 0.083	200 and 300±5	24 and 36±0.6w
	13—15	10.546 ± 0.102	8.100 ± 0.078	300±5	36±0.6w
	16—18	10.546 ± 0.102	8.100 ± 0.078	300±5	36±0.6w
	19—21	10.156 ± 0.104	7.800 ± 0.080	300 and 250±5	30 and 36±0.6w
	22—24	9.374 ± 0.108	7.200 ± 0.083	250±5	30w±0.6w
	25—27	9.374 ± 0.108	7.200 ± 0.083	250±5	30w±0.6w
	28—30	9.374 ± 0.108	7.200 ± 0.083	300 and 250±5	30w±0.6w
T4	1—3	8.203 ± 0.118	6.300 ± 0.091	200±5	24±0.6w
	4—6	8.203 ± 0.118	6.300 ± 0.091	200±5	24±0.6w
	7—9	8.203 ± 0.118	6.300 ± 0.091	200±5	24±0.6w
	10—12	8.984 ± 0.112	6.900 ± 0.086	200 and 250±5	24 and 30±0.6w
	13—15	9.374 ± 0.108	7.200 ± 0.083	250±5	30±0.6w
	16—18	9.374 ± 0.108	7.200 ± 0.083	250±5	30±0.6w
	19—21	9.765 ± 0.106	7.500 ± 0.081	250 and 300±5	30 and 36±0.6w
	22—24	10.546 ± 0.101	8.100 ± 0.078	300±5	36±0.6w
	25—27	10.546 ± 0.101	8.100 ± 0.078	300±5	36±0.6w
	28—30	10.546 ± 0.101	8.100 ± 0.078	300±5	36±0.6w

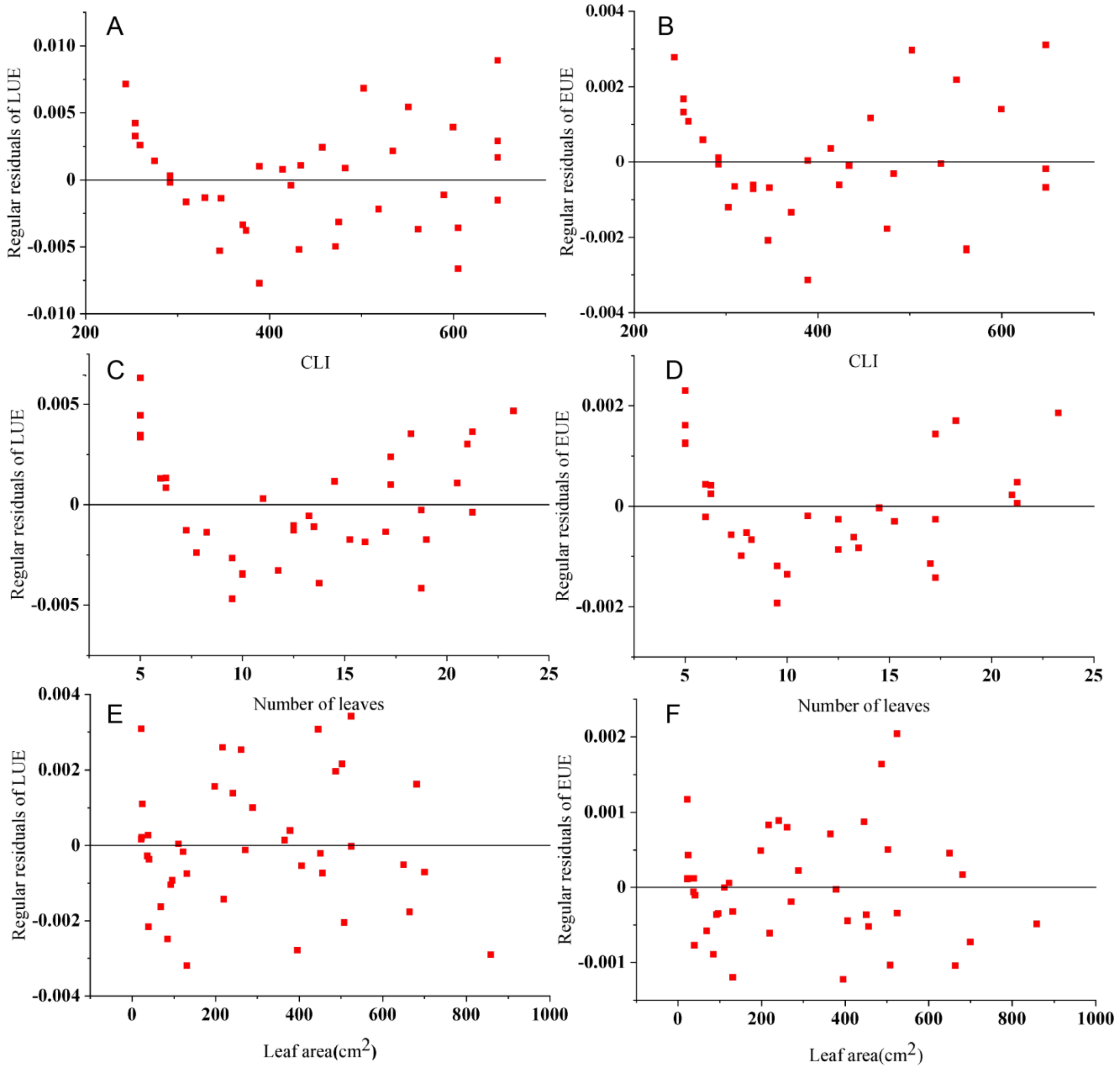

Figure S1. Regular residuals of the linear models for plant morphology, light environment, LUE, and EUE （A-F）.(A) CLI and LUE, (B) CLI and EUE, (C) Number of leaves and LUE, (D) Number of leaves and EUE, (E) Leaf area and LUE, (F) Leaf area and EUE.

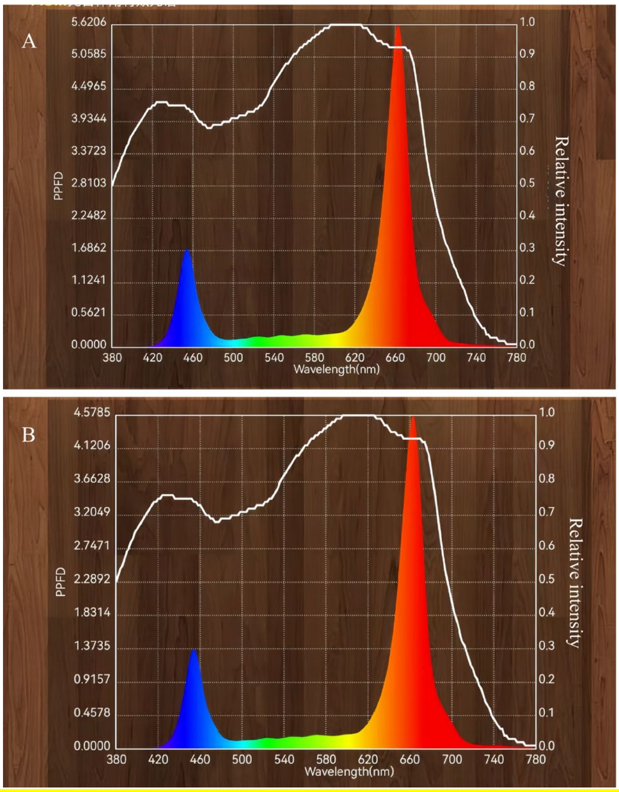

Figure S2. Different PPFD spectrum diagrams. (A) Spectrum at 250 μmol·m^{-2}·s^{-1}. (B) Spectrum at 200 μmol·m^{-2}·s^{-1}.
